# Production routes to bio-acetic acid: life cycle assessment

**DOI:** 10.1186/s13068-020-01784-y

**Published:** 2020-09-03

**Authors:** Erik Budsberg, Rodrigo Morales-Vera, Jordan T. Crawford, Renata Bura, Rick Gustafson

**Affiliations:** 1 School of Environmental and Forest Sciences, University of Washington, Seattle, Box 352100, WA 98195-2100 USA; 2 School of Agricultural and Forest Sciences, Catholic University of Maule, Center of Biotechnology of Natural Resources (CENBIO), Talca, Chile; 3 AECOM Corp, Richland, WA USA

**Keywords:** Acetic acid, Bioconversion, Biochemicals, Bioproducts, Biorefinery, Fossil fuel use, Global warming potential, Life cycle assessment

## Abstract

**Background:**

Similar to biofuels, numerous chemicals produced from petroleum resources can also be made from biomass. In this research we investigate cradle to biorefinery exit gate life cycle impacts of producing acetic acid from poplar biomass using a bioconversion process. A key step in developing acetic acid for commercial markets is producing a product with 99.8% purity. This process has been shown to be potentially energy intensive and in this work two distillation and liquid–liquid extraction methods are evaluated to produce glacial bio-acetic acid. Method one uses ethyl acetate for extraction. Method two uses alamine and diisobutyl ketone. Additionally two different options for meeting energy demands at the biorefinery are modeled. Option one involves burning lignin and natural gas onsite to meet heat/steam and electricity demands. Option two uses only natural gas onsite to meet heat/steam demands, purchases electricity from the grid to meet biorefinery needs, and sells lignin from the poplar biomass as a co-product to a coal burning power plant to be co-fired with coal. System expansion is used to account for by-products and co-products for the main life cycle assessment. Allocation assessments are also performed to compare the life cycle tradeoffs of using system expansion, mass allocation, or economic allocation for bio-acetic acid production. Finally, a sensitivity analysis is conducted to determine potential effects of a decrease in the fermentation of glucose to acetic acid.

**Results:**

Global warming potential (GWP) and fossil fuel use (FFU) for ethyl acetate extraction range from 1000–2500 kg CO_2_ eq. and 32–56 GJ per tonne of acetic acid, respectively. Alamine and diisobutyl ketone extraction method GWP and FFU ranges from −370–180 kg CO_2_ eq. and 15−25 GJ per tonne of acetic acid, respectively.

**Conclusions:**

Overall the alamine/diisobutyl ketone extraction method results in lower GWP and FFU values compared to the ethyl acetate extraction method. Only the alamine/diisobutyl extraction method finds GWP and FFU values lower than those of petroleum based acetic acid. For both extraction methods, exporting lignin as a co-product produced larger GWPs and FFU values compared to burning the lignin at the biorefinery.

## Background

There is significant interest in converting lignocellulosic biomass to biofuels with the goal of producing transportation fuels that reduce greenhouse gas emissions compared to petroleum based fuels. Many of these studies assessed the feasibility of converting this biomass to ethanol via fermentation methods [1–3]. Life cycle assessments have been performed to assess the environmental impacts of producing ethanol. Results from these LCA studies report that while there is variability amongst the biofuel production methods related to feedstock type and fermentation method, ethanol biofuel will generally have a lower global warming potential lower than gasoline [2–4].

Similar conversion processes may be used to produce biochemicals which can displace petroleum based chemicals. Some of the chemicals recently proposed to be produced from lignocellulosic biomass include: polylactic acid [5–8], polyethylene [5], polyethylene terephthalate [8], polyhydroxyalkanote [6, 7], succinic acid [9], butanediol [10] and starch based polymers [6, 7]. LCA results on the production and use of these products differ, but in general bio-based chemicals are found to be superior in regards to lower global warming potentials compared to the petroleum based fuels they intend to replace [7]. Main sources of variation between biochemical LCAs can be traced to the source of electricity used in manufacturing, system boundaries, and allocation methods when more than one product is made during biochemical production [7].

A common issue in biochemical LCA studies is how to set the system boundaries [7]. Biofuel LCAs typically include full cradle to grave analysis. The use/disposal of fuel is easy to identify (combustion) and emissions resulting from this can be readily tracked. Use and disposal of chemicals, especially platform chemicals, is much less straightforward. These chemicals are typically used in the synthesis of other chemicals. The use and disposal of these chemicals varies and can include large and/or undefined temporal scopes [6, 8, 11]. Addressing these issues can be complex and end of life scenarios can introduce uncertainty and variability in LCAs [6, 11]. To avoid end of life issues some researchers choose to set cradle to biorefinery gate system boundaries that include the manufacturing of the biochemical(s), but not use or disposal [6]. Others have performed full cradle to grave assessments by making assumptions of the use and disposal of the biochemical(s) [5, 6, 8]. Those that include end of life scenarios found that it can have a significant effect on the global warming potential results, but these results are highly variable [6].

In this research, the petroleum based chemical targeted for replacement by a biochemical is acetic acid. Currently acetic acid is primarily made by methanol carbonylation [12]. The process works by reacting methanol with carbon monoxide in the presence of a metal carbonyl catalyst (Cativa process) [13]. Annual worldwide acetic acid demand in 2013 was 13.15 million tonnes and is expected to reach 17.3 million tonnes [14]. Acetic acid has a wide range of potential end uses and can be used to make products such as paints, plastics, adhesives, and food [12]. Carbonylation of methanol to produce acetic acid has a cradle to gate global warming potential of 1 kg CO_2_ eq./kg of acetic acid [15] and manufacturing acetic acid, therefore, contributed approximately 13.15 million tonnes of CO_2_ eq. to the atmosphere in 2013. Reducing the GWP of acetic acid production would be a positive step in mitigating the effect of chemical production on climate change.

Bio-based acetic acid can be produced from lignocellulosic biomass via a bioconversion process. Following pretreatment and hydrolysis an acetogen can be used in the fermentation step to produce acetic acid. Acetogens only produce acetic acid as they digest sugars and have a theoretical yield of 100% [16]. To be widely marketable, acetic acid must be distilled to glacial purity (99.8% acetic acid). Distillation of acetic acid to glacial purity can be difficult if it is not in high concentration when fed to the recovery process [12]. Preliminary analysis in our research identified direct distillation as a major environmental and economic hurdle to producing bio-based acetic acid. Two potentially viable recovery processes for bio-based acetic acid have been proposed and are evaluated on a life cycle basis in this research. The first uses ethyl acetate extraction (EAX) to extract acetic acid from water. The second uses an alamine and diisobutyl ketone (DIBK) solvent to extract the acetic acid. Alamine/DIBK extraction is abbreviated as ADX within the text.

In addition to assessing the two extraction methods this work also seeks to identify life cycle tradeoffs in meeting biorefinery energy demands. Steam to operate the biorefinery can come from burning lignin supplemented with natural gas or purely from natural gas. Burning lignin has the advantage of lowering the GWP of the process and potentially producing renewable electricity which can be exported to further reduce the GWP (Borrion et al. 2012, Budsberg et al. 2015). The disadvantage to this approach is the high capital cost of a high pressure boiler that can burn lignin. Alternatively the lignin may be sold for co-combustion in a coal fired plant [17]. If lignin is exported, only natural gas would be used to produce the necessary steam for the biorefinery. This process would have a much lower capital cost but the GWP may be higher as fossil fuel use is increased at the biorefinery. To address this concern life cycle comparisons of burning lignin onsite vs exporting it as a co-product are included in this research.

Four biorefinery designs are assessed in this research: EAX used to extract acetic acid with lignin and natural gas combusted onsite to provide steam (EAX OC), EAX used to extract acetic acid with natural gas burned onsite to provide steam and lignin exported to be co-fired with coal at a coal burning power plant (EAX LE), ADX used to extract acetic acid with lignin and natural gas combusted onsite to provide steam (ADX OC), and ADX used to extract acetic acid with natural gas burned onsite to provide steam and lignin exported to be co-fired with coal at a coal burning power plant (ADX LE). In the onsite combustion scenarios steam produced from burning lignin and natural gas is passed through a turbine to also produce electricity. Electricity generated from this process exceeds onsite demands and excess electricity is sold as byproduct of acetic acid production. In the lignin exporting scenarios only natural gas is burned at the biorefineries to meet steam demands, and capital costs are minimized using a lower pressure boiler and no turbine to produce electricity. Electricity needed to operate these biorefineries is assumed to be purchased from the grid.

Cradle to biorefinery exit gate system boundaries are used to evaluate acetic acid production. System expansion is used as the primary method to deal with either the production of an excess electricity by-product (when combusting lignin and natural gas onsite at the biorefineries) or the lignin co-product (when lignin is exported to a coal burning power plant). A functional unit of 1 tonne of acetic acid produced from a biorefinery system with 21 year operating time frame is used in the analysis. Environmental impacts considered are the 100 year Global Warming Potential (GWP) [18], and Fossil Fuel Use (FFU). Scenario results are compared to each other as well as to petroleum based acetic acid produced by methanol carbonylation [15]. In addition to the four main scenarios described above, both an allocation assessment and a sensitivity analysis are conducted. The allocation assessment looks at the life cycle effects of the lignin co-product (lignin exporting scenarios) using both economic and mass allocation approaches. The sensitivity analysis tests the effect that the acetic acid yield has on life cycle impacts.

The work presented here is part of an investigation into the environmental and economic impacts of bio-acetic acid commercially produced via an advanced bioconversion pathway. The acetic acid is produced using a biorefinery design similar to the one reported by Crawford et al. [19] and Budsberg et al. [20] to produce hydro-carbon bio-jet fuel. The work is part of a collaboration between industry, government, and academia to develop a sustainable biofuels and biochemicals industry. In this article LCA is used to determine potential environmental impacts of bio-acetic acid production.

## Results

The life cycle assessment is broken up into the following categories to identify areas within the system boundaries that contribute to environmental impact categories:

- Carbon in biomass: CO_2_ absorbed by photosynthesis in harvested chips, above ground and below ground stumps, and coarse roots over a 21 year time horizon.
- Poplar growth and harvesting: All technosphere processes associated with growing and harvesting poplar. Includes direct land use change emissions.
- Ancillary chemicals: All chemicals and inputs required for biorefinery operations, including natural gas.
- Purchased electricity: Electricity needed for biorefinery operations in scenarios, where lignin is exported to coal fired power plants.
- Transportation: Includes transportation of poplar from farm to biorefinery gate, transportation associated with all chemical inputs, and transportation of lignin to coal plant (co-product scenarios).
- Biorefinery: All operations performed, raw materials used, and emissions from the biorefinery.
- Lignin at power plant: Emissions resulting from the combustion of lignin at a coal power plant (lignin co-product exporting scenarios).
- Avoided production: In the EAX OC and ADX OC scenarios an avoided production credit is earned from displacing electricity produced from natural gas with excess electricity produced at the biorefinery. In the EAX LE and ADX LE scenarios an avoided production credit is earned from displacing coal with lignin at a coal power plant.
- Petro-acetic acid: Cradle to refinery exit gate life cycle impacts for production of petroleum based acetic acid.

### Global warming potential

**Fig. 1:**
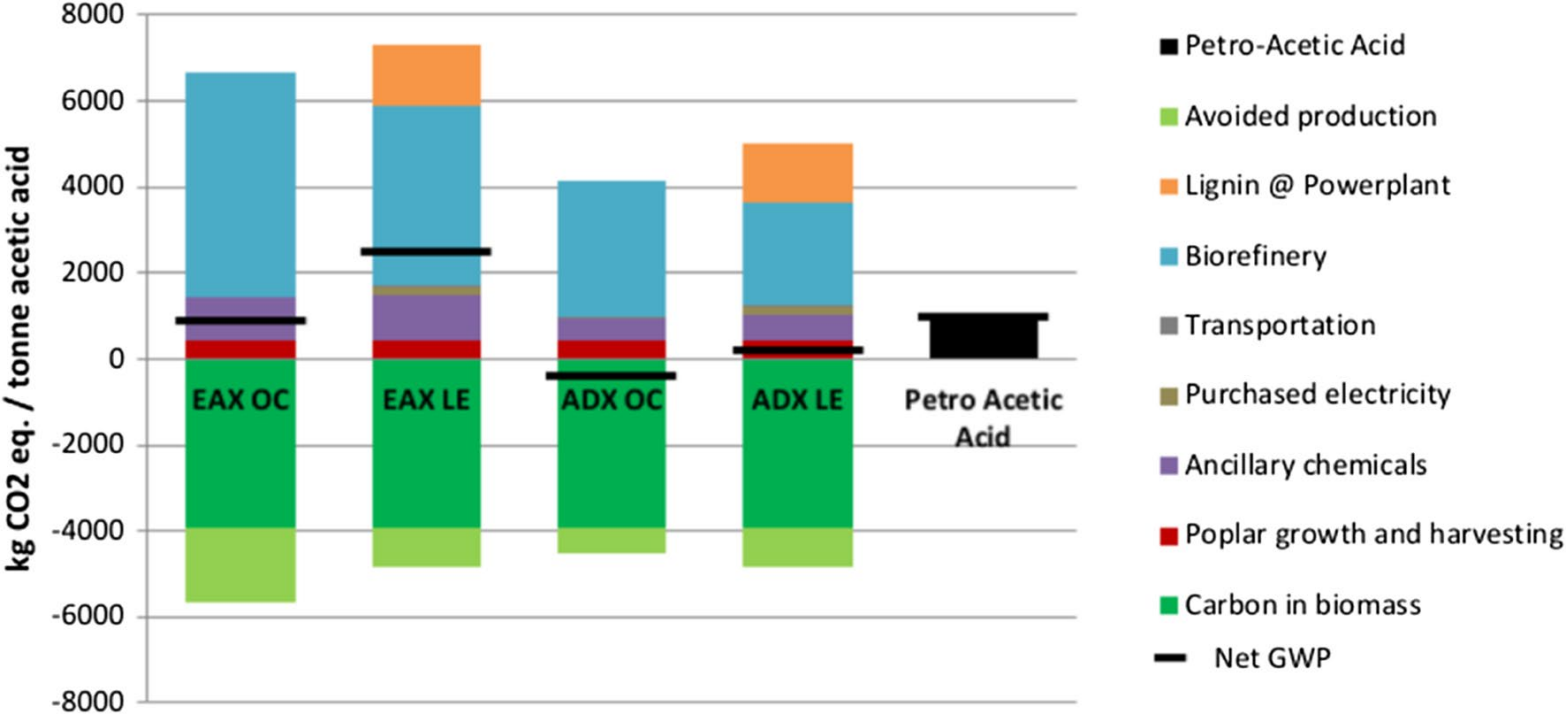
Global warming potentials of acetic acid production scenarios

The GWPs calculated from each bio-acetic acid production scenario and petroleum based acetic acid are presented in Fig. 1. Net GWPs are listed in Table 1. For all scenarios, the biorefinery section is the largest source of greenhouse gases (GHGs) contributing to the GWP. Lignin combusted at the power plant is the second largest contributor to the GWP in the EAX LE and ADX LE scenarios. A significant proportion of the GHGs contributing to the GWP from the biorefinery operations, and all the GHGs from lignin combustion at the power plant, are from combustion of biomass and considered biogenic. The ancillary chemicals category is the second largest contributor to the GWP in the EAX OC and ADX OC scenarios, and third largest in the lignin export scenarios. All emissions contributing to the GWP from the ancillary chemicals category are from the use of fossil fuels and considered non-biogenic. Across all scenarios poplar growth and harvesting follows the ancillary chemicals category as the next largest contributor of GHGs to the GWP. Top sources of GHGs to the GWP from poplar growth and harvesting are direct land use change (276 kg CO_2_ eq. per tonne of acetic acid) followed by nitrogen fertilizer production and N_2_O emissions from its use (35 kg CO_2_ eq. per tonne of acetic acid, combined), and diesel use in farm equipment (30 kg CO_2_ eq. per tonne of acetic acid). Transportation and purchased electricity comprise only small percentages of the GWPs. The avoided production credit reduces the GWP of each scenario. In the EAX OC and ADX OC this credit is generated by exporting excess electricity and displacing electricity produced from natural gas. In the EAX LE and ADX LE scenarios the avoided production is for directly displacing coal at a coal burning facility with lignin. The type of fossil fuel displaced has a notable effect on the avoided production credit generated. For every MJ of natural gas electricity that is displaced a credit of 0.19 kg of CO_2_ eq. is earned. For every MJ of coal electricity displaced a credit of 0.32 kg of CO_2_ eq. is earned. Per tonne of acetic acid, the avoided production credit decreases the CO_2_ eq. of the net GWPs of EAX OC, EAX LE, ADX OC, and ADX LE by 1800 kg, 915 kg, 590 kg and 915 kg, respectively. The effect of the avoided production credit on the life cycle impacts of acetic acid production are evaluated below in the allocation section of the results.

**Table 1:** Global warming potential and fossil fuel use results per tonne of acetic acid

Scenario	Net GWP CO_2_ eq. (kg t^−1^)	Net FFU (GJ t^−1^)
EAX OC	1000	32
EAX LE	2500	56
ADX OC	−370	15
ADX LE	180	25
Petro-acetic acid	1000	44

Net GWPs are scenario dependent, but overall the EAX GWPs are higher than the ADX GWPs (Table 1, Fig. 1). Compared to petroleum based acetic acid EAX OC has an equivalent GWP, while the GWP of EAX LE is 150% higher. The GWPs of ADX OC and ADX LE are 140%, and 82% lower than petro-acetic acid, respectively. For both EAX and ADX, the onsite lignin combustion scenarios achieve the lowest GWPs.

**Fig. 2:**
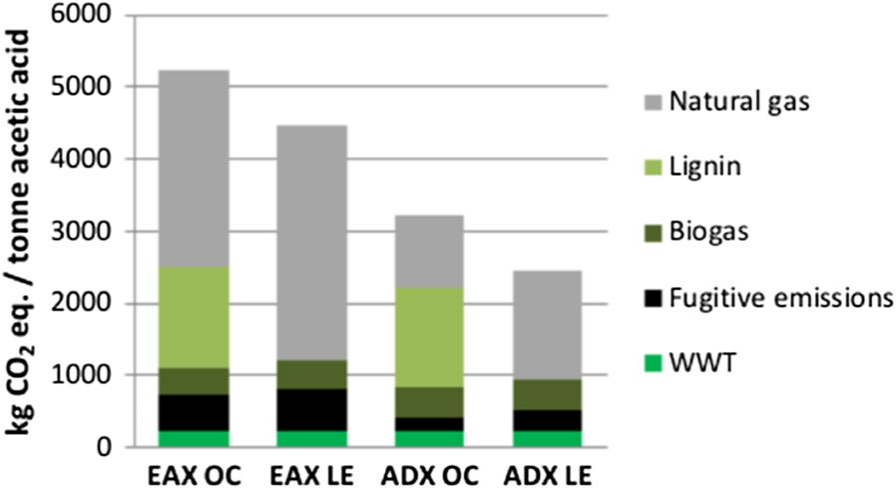
Sources contributing to the global warming potential of the biorefinery in each scenario. Global warming potentials for natural gas, lignin, and biogas are from the combustion of each fuel. Fugitive emissions are natural gas emissions that are assumed to leak/escape from various stages of biorefinery operations. Wastewater treatment (WWT) includes CO_2_ emissions for both anaerobic digestion and aerobic digestion. Anaerobic digestion produces CH_4_ which is sent to the boiler for combustion (and ultimately emitted as CO_2_)

A breakdown of sources of GHGs within the biorefineries is presented in Fig. 2. The two primary GHGs emitted from the biorefinery simulations are CO_2_ and CH_4_. CO_2_ is emitted from the combustion of lignin, natural gas, biogas, and the aerobic stage of the wastewater treatment process. Fugitive CH_4_ emissions result from small leaks of natural gas within the biorefinery system. In all scenarios natural gas combustion is the main non-biogenic source of greenhouse gases contributing to the GWP. In the EAX OC and ADX OC onsite lignin combustion is the second largest contributor to the GWP. A significantly higher natural gas use in the EAX scenarios compared to the ADX scenarios results in higher fugitive methane emissions at the biorefinery. Fugitive methane emissions are the second largest source of GHGs contributing to the GWP at the EAX LE biorefinery. In the ADX LE scenario, emissions from the combustion of biogas generated from the anaerobic stage of the wastewater treatment process is the second largest source of GHGs contributing to the GWP. CO_2_ emissions from the aerobic stage of the wastewater treatment process are similar in all four scenarios.

**Fig. 3:**
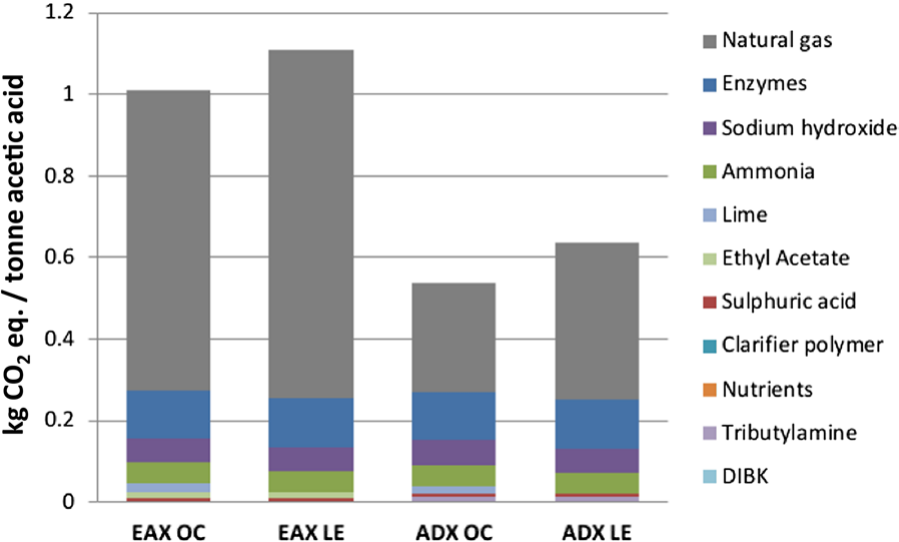
Sources contributing to the global warming potential of the ancillary chemicals category in each scenario

A breakdown of the GWP in the ancillary chemicals category is shown in Fig. 3. In all scenarios the largest contributor to the GWP is natural gas production and acquisition followed by the manufacturing of enzymes, sodium hydroxide, and ammonia. The GWP effect of natural gas acquisition is more than two times greater in the EAX scenarios than in the ADX scenarios. This is a result of the ethyl acetate extraction method demanding a higher heat/steam input than alamine/DIBK extraction. All other major chemical inputs and their GWP effect are the same across all scenarios (except for lime which is not required in lignin exporting scenarios as lime is used to remove sulfur emissions from combusting biomass–a process not performed in the biorefinery in the these scenarios). Both ethyl acetate and alamine/DIBK extractive chemicals have little effect on the GWP owing to high recycling rates within their respective biorefineries.

### Fossil fuel use

**Fig. 4:**
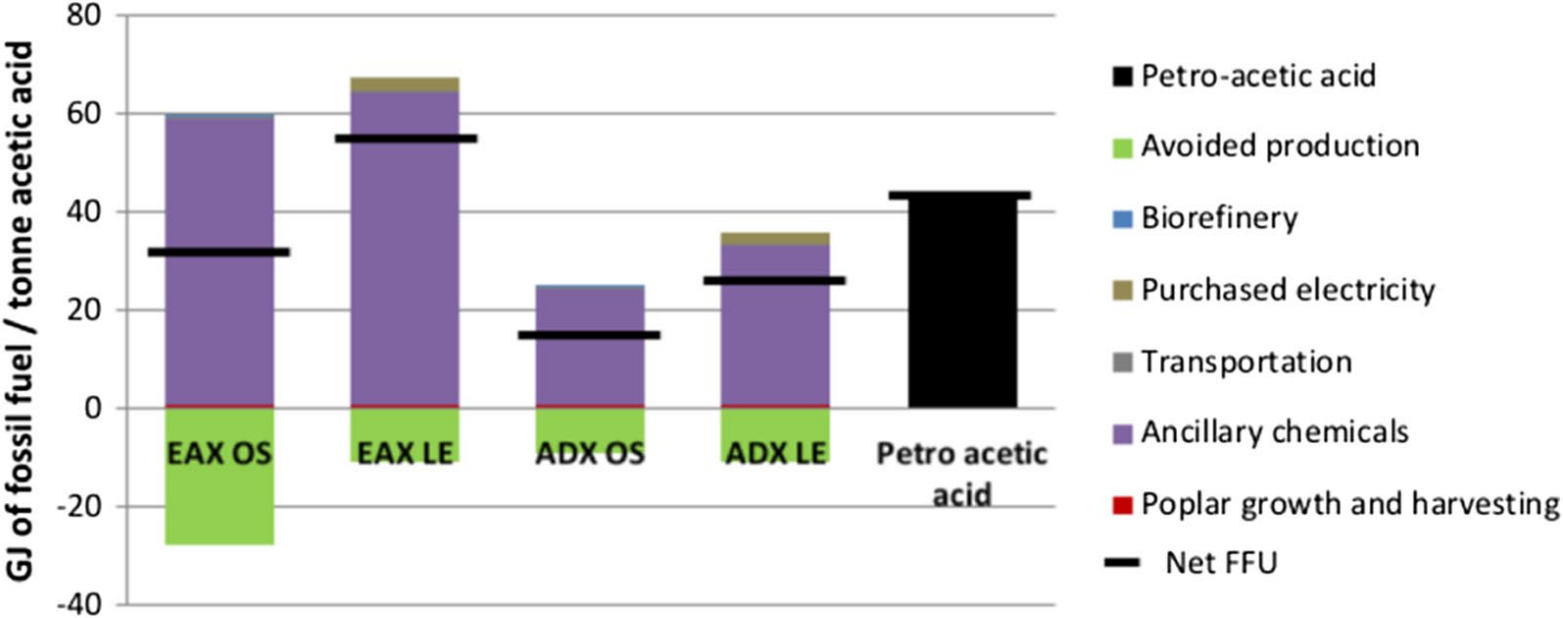
Fossil fuel use for each acetic acid production scenario

FFU values were calculated for bio-acetic acid and petro-acetic acid production scenarios and are presented in Fig. 4. Net FFU values are listed in Table 1. Compared to petro-acetic acid EAX LE uses 28% more fossil fuels, while EAX OC, ADX OC, and ADX LE use 28%, 65%, and 43% less fossil fuels, respectively. The acquisition/manufacturing of products within the ancillary chemicals category is responsible for the majority of all fossil fuel use. Purchased electricity, transportation and poplar growth and harvesting are credited with only a minor amount of fossil fuel use. Avoided production credits reduce net fossil fuel use. Avoided production of marginal electricity production (natural gas) reduces FFU in EAX OC by 27 GJ t^−1^ and in ADX OC by 9.3 GJ t^−1^. Avoided production of coal electricity reduces FFU in EAX LE and in ADX LE by 11 GJ t^−1^.

**Fig. 5:**
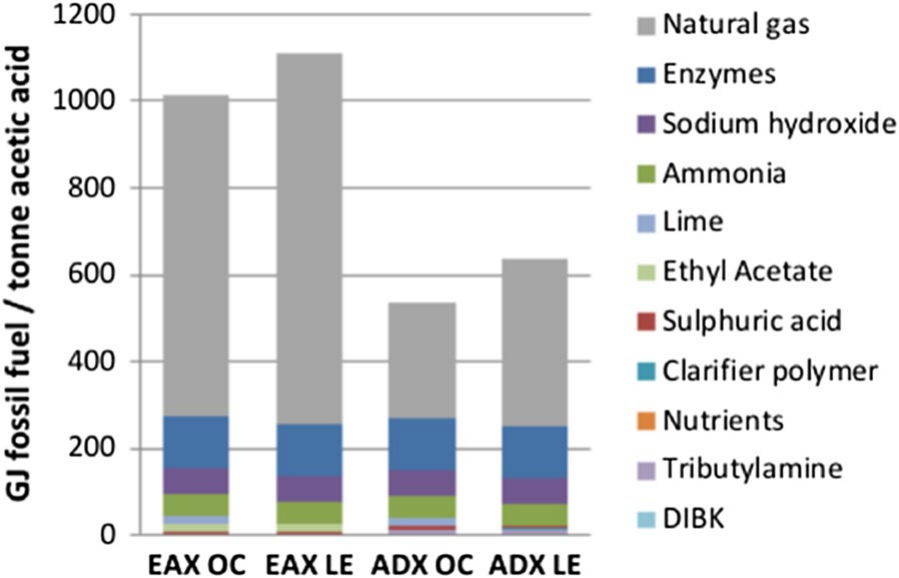
Fossil fuel use within the ancillary chemicals category of each scenario. Included in the calculations are the amount of fossil fuels needed to extract/produce/transport each chemical. For natural gas, this also includes the embodied fossil fuel energy in the gas itself

For all scenarios natural gas acquisition/production is the largest user of fossil fuels within the ancillary chemicals category (Fig. 5). Per tonne of acetic acid, natural gas FFU used in the production of acetic acid in the EAX OC, EAX LE, ADX OC, and ADX LE scenarios is 54 GJ, 63 GJ, 20 GJ, and 28 GJ, respectively. Manufacturing of enzymes is the second largest consumer of fossil fuels and is responsible for 1.8 GJ t-^1^ of acetic acid. All other products within the ancillary chemicals category combine to use approximately 2.4 GJ t^−1^ of acetic acid and individually represent 4% to 10% of the total fossil fuels used to make acetic acid.

### Allocation and sensitivity analysis

Results of the allocation analysis are presented in Table 2. For both the EAX LE and ADX LE mass allocation assessments 69.5% of the life cycle impacts are attributed to acetic acid production and 30.5% of the impacts to the lignin co-product. Using economic allocation 95.5% and 94.6% of the impacts are attributed to acetic acid production in the EAX LE and ADX LE scenarios, respectively. The small difference in the economic allocation is due to the ADX scenarios having a calculated lower minimum selling price for acetic acid. Compared to the system expansion approach economic allocation results in higher GWP and FFU values for the EAX LE and ADX LE acetic acid life cycle scenarios. The effect of mass allocation is not as significant as economic allocation. In the case of EAX LE mass allocation actually reduces the net GWP and FFU compared to the system expansion approach. For ADX LE mass allocation results in an increase to the net GWP and no change to the net FFU compared to the system expansion approach.

**Table 2:** Global warming potential and fossil fuel use allocation results

Scenario	Net GWP CO_2_ eq. (kg t^−1^)	Net FFU (GJ t^−1^)
EAX LE: system expansion	2500	56
EAX LE: economic allocation	3200	64
EAX LE: mass allocation	1900	47
ADX LE: system expansion	180	25
ADX LE: economic allocation	960	34
ADX LE: mass allocation	310	25

Results are listed per tonne of acetic acid

Sensitivity analysis results are presented in Table 3. Decreasing the glucose to acetic acid fermentation yield by 10% decreases the amount of acetic acid produced per tonne of biomass from 533 to 482 (~ 10%). Decreasing the acetic acid yield does not change the rate of inputs to the biorefinery. Therefore, relative to a tonne of acetic acid, inputs to the biorefinery (ancillary chemicals) increase as acetic acid yield drops (i.e., same amount of inputs, but a lower final product yield). Inputs to the biorefinery increase by 10% to 11%, except for natural gas use which only increases by 6%. Natural gas use does not increase as much as the other inputs within the ancillary chemicals category as a decrease in the conversion of glucose to acetic acid results in more biomass available to convert to bioenergy and less acetic acid to recover. The increase in available biomass for bioenergy also increases the amount of excess electricity produced by approximately 6%. Combustion of the additional biomass increases biogenic CO_2_ emissions at the biorefinery by 19%. The total effect of decreasing the acetic acid yield, the relative increase in ancillary chemicals, and an increase in excess electricity are increases to the GWP and FFU of EAX OC and EAX LE. EAX OC GWP and FFU increase by 20% and 6.3%, respectively. EAX LE GWP and FFU increase by 12% and 7.1%, respectively.

**Table 3:** Global warming potential and fossil fuel use sensitivity analyses

Scenario	Net GWP CO_2_ eq. (kg t^−1^)	Change from base case (%)	Net FFU (GJ t^−1^)	Change from base case (%)
EAX OC: −10% Fermentation yield	1200	20	34	6.3
EAX LE: −10% Fermentation yield	2800	12	60	7.1

## Discussion

Poplar biomass can be used to produce acetic acid that has a lower GWP and uses less fossil fuels than petroleum based acetic acid produced via carbonylation; however, it depends on the extraction method used to concentrate and purify the acetic acid (Table 1). EAX OC results in lower FFU and an equivalent GWP compared to petroleum acetic acid. EAX LE has a GWP and FFU significantly higher than petro-acetic acid. On the other hand, ADX OC and ADX LE both have much lower GWPs and FFU values compared to petro-acetic acid. All poplar based acetic acid production scenarios benefit from large avoided production credits. The avoided production credits help to lower the net GWP and FFU for each scenario. Without the credits GWP and FFU values would be much larger, and when removed, ADX OC is the only scenario that would still have a GWP and FFU lower than petro-acetic acid.

Between the two extraction methods investigated here, the alamine/DIBK extraction method to produce acetic acid via bioconversion of poplar biomass achieves the lowest GWPs and FFU values. Compared to the EAX scenarios, ADX scenarios achieve GWP CO_2_ eqs. that are 1370 kg t^−1^ lower when burning lignin onsite and 2320 kg t^−1^ lower when lignin is exported to a coal power plant. For FFU ADX values are 17 GJ t^−1^ lower when burning lignin onsite and 31 GJ t^−1^ lower when lignin is exported to a coal power plant. ADX scenarios show lower net GWPs and FFU values than the EAX platform, because the ADX pathway requires less heat/steam and, therefore, less natural gas (Table 5, Figs. 1 and 4). Natural gas use is 34 MJ lower in ADX OC vs EAX OC and 33 MJ lower in ADX LE vs EAX LE.

EAX OC has a lower GWP and FFU than EAX LE (Table 1). Drivers for this difference include changes to natural gas use, electricity purchasing, and the avoided production credit. The EAX OC biorefinery design includes a turbine onsite so that high pressure steam created from the combustion of lignin and natural gas can be used to produce electricity. In the EAX LE scenario lignin is exported to a coal burning facility and biorefinery capital expenses are decreased using a lower pressure boiler and foregoing a turbine; therefore, no electricity is produced onsite and more low pressure steam must be generated for heating needs. To make up for the loss in energy by exporting the lignin the EAX LE biorefinery must use more natural gas and purchase electricity, thereby increasing use of fossil fuel based resources compared to EAX OC. The decision to use a low pressure boiler and to not produce electricity onsite in the EAX LE scenario also results in lower thermal energy generation compared to EAX OC. In EAX OC electricity is not only produced from biomass and biogas, but also from steam produced from natural gas combustion. The total amount of excess electricity that can be used to displace electricity production elsewhere (and counted as an avoided production credit) is, therefore, larger than in EAX LE when just lignin is used to displace coal. It was originally hypothesized that EAX LE would result in a lower GWP, because on a per unit of energy basis, coal-based electricity has a larger GWP than natural gas based electricity. It was assumed that this would result in an avoided production credit for EAX LE that would out-compete the loss in total energy production. The additional low carbon electricity produced in the EAX OC scenario, however, outweigh the benefits of coal displacement in the EAX LE scenario.

Similar to the EAX scenarios, ADX OC scenario finds a lower GWP and FFU than ADX LE. The demand for increased biorefinery natural gas use and purchased electricity in ADX LE help to drive up both the GWP and FFU. Unlike the EAX scenarios; however, the avoided production credit is greater in the lignin exporting model. ADX OC does not produce as much electricity as EAX OC, and therefore, there is not a significant loss in energy produced when comparing ADX OC to ADX LE. The increased avoided production credit in ADX LE results from the shift to displacing coal-based electricity instead of natural gas based electricity. Even with the greater avoided production credit; however, the ADX OC scenario has lower GWP and FFU values. The difference between the GWP and FFU values of ADX OC and ADX LE are not as large as the differences between EAX OC and EAX LE (Table 1).

Avoided production credits are identified as a significant benefit to the acetic acid production scenarios. Economic and mass allocation assessments were performed to determine the life cycle impacts associated with just acetic acid by removing the environmental burdens and benefits of the lignin co-product from the life cycle of acetic acid production and to compare environmental impacts using different LCA methodology. The economic value of lignin is small compared to acetic acid and based on economic allocation almost all life cycle process (except avoided production, because it is downstream of acetic acid production) are attributed to acetic acid production. Mass allocation shares the environmental burdens between lignin and acetic acid more evenly with ~ 30% attributed to the lignin co-product and ~ 70% attributed to acetic acid. Economic allocation produces higher GWPs and FFU than mass allocation as more of the life cycle processes are associated with acetic acid production. In both economic and mass allocation displacement of coal by the lignin co-product is completely removed from acetic acid production. This process occurs downstream of the separation of lignin from the acetic acid production process and is, therefore, entirely attributed to the lignin co-product. For economic allocation, removal of the avoided production credit without the additional removal of environmental burdens from acetic acid life cycle results in large increases to the EAX LE and ADX LE GWPs and FFU values (Table 2). The effect of mass allocation has on the GWP and FFU differs for EAX LE and ADX LE. Although mass allocation removes the benefit of the avoided production credit it also removes 30% of the environmental burdens from the respective acetic acid life cycles. For EAX LE this means the GWP and FFU are reduced compared to that obtained using system expansion. For ADX LE the GWP increases, but not as much as it does for economic allocation and FFU remains the same as the system expansion model. The reason for the GWP increase in ADX LE is the loss of the avoided production credit. In the ADX LE system expansion model the avoided production credit is relatively much larger to the overall GWP than it is for EAX LE (Fig. 1). The loss of the avoided production credit when using an allocation approach is, therefore, much more pronounced. Even though 30% of the environmental burdens are removed from the acetic acid life cycle in the mass allocation model, it is not enough to make up for the loss in avoided production. The results of the allocation analysis indicate that for both EAX LE and ADX LE life cycle results can greatly vary depending on acetic acid production design and the allocation methodology. This further underscores the importance of transparency in LCAs, as choosing one methodology over another can significantly change the results of a study.

Sensitivity analyses were conducted to evaluate the effect of a varied acetic acid fermentation yield. Decreasing the fermentation yield of glucose to acetic acid by 10% increased the EAX OC and EAX LE GWP by 20% and 12%, respectively (Table 3). There are two driving factors in the increase to GWP. First, decreasing the fermentation yield decreases acetic acid production, but requires roughly the same amount of inputs (feedstock and ancillary chemicals). Relative to a tonne of acetic acid the life cycle impacts associated with these impacts increases. Secondly, decreasing the acetic acid yield decreases the amount of carbon stored in the acetic acid end product and increases the amount carbon released through combustion of unfermented carbohydrates. The increase of inputs required to make a tonne of acetic acid combined with less carbon stored in the final products results in a “double hit” to the GWP. In contrast EAX OC and EAX LE FFU only increase by 6.3% and 7.1%, respectively, because of the decreased fermentation yield. FFU values are not affected by stored carbon (or the lack thereof) and only the increase in inputs relative to acetic acid production is factored into its calculations. Additionally, in relation to the other biorefinery inputs, the increase in available biomass for bioenergy reduces the demand for natural gas. Natural gas is the largest contributor to FFU and identifying non fossil fuel based replacements for natural gas will be key to reducing the process sensitivity (and overall FFU) to yield.

Currently there are no LCAs for production of bio-acetic acid in the literature to make direct comparisons too. An approximate comparison can be made to a process to produce succinic acid from corn stover using an LLE technique [9]. The bio-succinic production process uses the pretreatment and hydrolysis process proposed by Humbird et al. [1] to release sugars for fermentation (same as in the bio-acetic acid process). The released sugars are fermented using yeast (*Actinobacillus succinogenes)* to produce succinic acid, and the succinic acid is extracted using tri-n-actylamine in an LLE column. Similar to the bio-acetic acid research presented here, natural gas use was found to be one of the largest sources of GHGs and fossil fuel use (26.6 GJ of natural gas used per tonne of succinic acid produced). GWP and FFU are found to be 692 kg CO_2_ eq. and 25.2 GJ per tonne of bio-succinic acid, respectively. The GWP and FFU for bio-succinic acid are lower than those calculated for the EAX scenarios and higher or equivalent than the GWP and FFU calculated for ADX scenarios. Similar to the ADX scenarios, bio-succinic acid GWP and FFU values are significantly lower than their petroleum based counterparts [9].

## Conclusion

Both ethyl acetate (EAX) and alamine/DIBK (ADX) are identified as viable extraction methods to produce glacial acetic created from the bioconversion of poplar biomass. The acetic acid produced from this process is identical to acetic acid produced from petroleum and can serve as a bio-based alternative without a loss in product quality. A goal in developing a bio-acetic acid and testing different extraction methods is to identify processes that reduce the GWP and FFU compared to petro-acetic acid. Only the ADX method is able to achieve these goals. EAX would result in a GWP that is either equivalent to or higher than that of petro-acetic acid. Natural gas use is a major contributor to the GWP and FFU for both extraction methods. The ability of the ADX method to operate with lower steam demands, and, therefore, use less natural gas, is a major factor in its overall lower GWP and FFU values. Identifying effective methods to reduce or replace natural gas with a renewable will reduce environmental impacts in the production of acetic acid.

Using lignin onsite or exporting it for co-firing at coal power plant can also have a significant effect on GWP and FFU values. Choosing between onsite lignin combustion and exporting it off site will affect the avoided production credit, natural gas use, and the need to purchase electricity. Allocation analysis identified that removing avoided credits from the system boundaries of acetic acid production generally increases the GWP and FFU. Additionally, environmental impacts associated with acetic acid production are identified as being sensitive to changes in acetic acid fermentation yield. Future research should focus on how to maintain a high acetic acid yield to avoid reducing the environmental benefits producing bio-acetic acid.

## Methods

In this study the production of acetic acid via the bioconversion of poplar biomass is evaluated using life cycle analysis. Models of acetic acid production plant with an annual biomass processing of 227,000 BDT/year were simulated in ASPEN-Plus chemical engineering modeling software, producing 120,650 tonnes per year of acetic acid for EAX and ADX solvent based scenarios. Total capital expenses were estimated at 245, 197, 223 and 187 million USD for EAX OC, EAX LE, ADX OC, and ADX LE, respectively. Scenarios are assessed that measure the life cycle environmental tradeoffs between acetic acid distillation/extraction methods, and within these models looking at burning lignin onsite or using the lower capital cost approach of selling the lignin to a coal power plant. Cradle to biorefinery gate system boundaries are set for acetic acid production to include the growth and harvesting of poplar biomass, biorefinery operations, and manufacturing of all necessary inputs (i.e., process chemicals, energy). Use and disposal of acetic acid is beyond the scope of this study. Environmental impacts to be assessed include the global warming potential, and fossil fuel use.

**Fig. 6:**
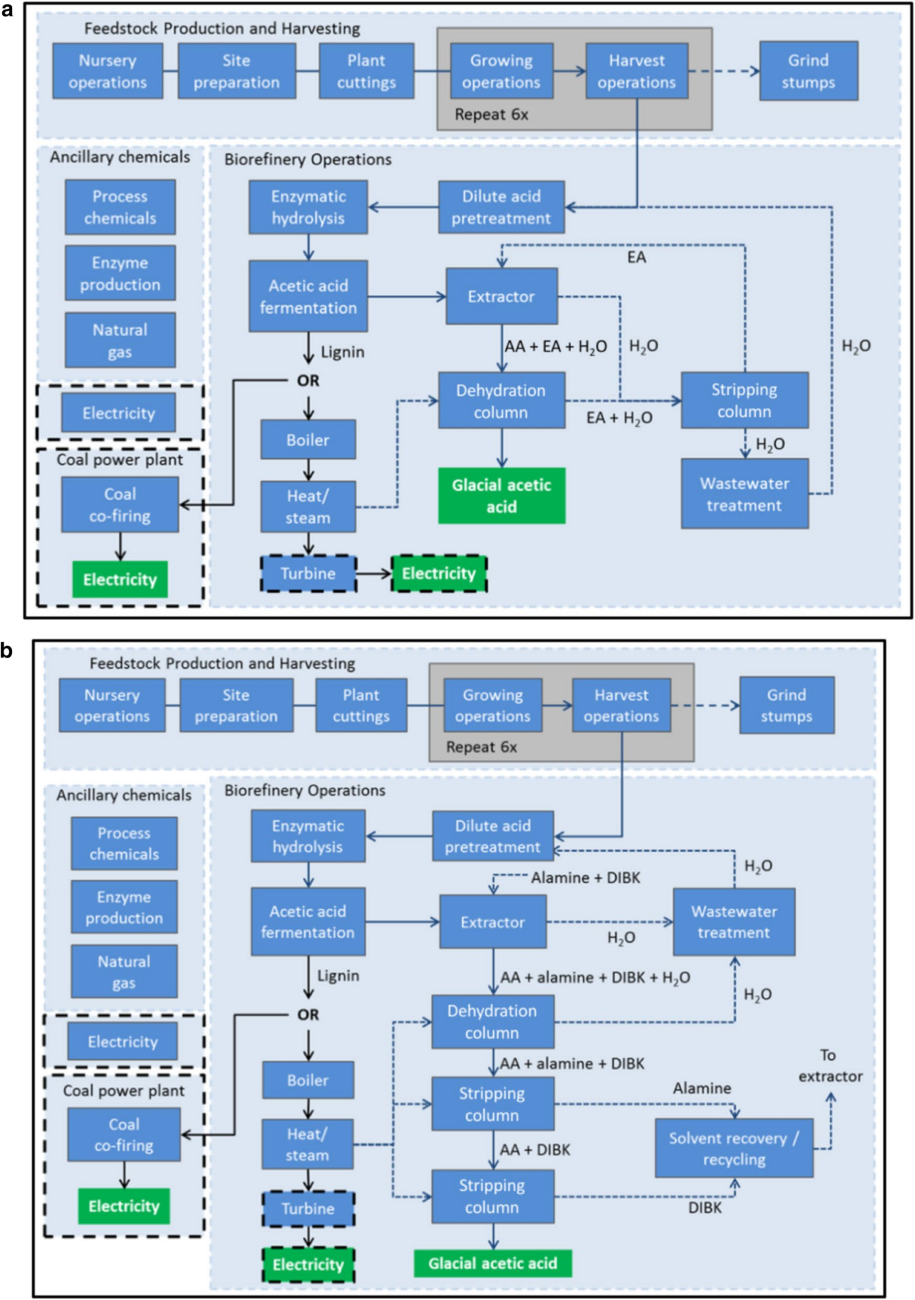
**a** Acetic acid (AA) extracted and distilled using ethyl acetate (EA). Both lignin scenarios are represented in the system boundaries figure. Black dashed line boxes indicate lignin scenario dependent operations. Lignin can either be burned onsite in the boiler to help produce heat/steam/electricity or sold to a coal power plant and co-fired with coal to produce electricity. If lignin is burned onsite, steam is run through a turbine to produce electricity. If lignin is exported to a coal power plant, no onsite electricity is made and electricity must be purchased from the grid for biorefinery operations. Green boxes highlight product made/energy produced.** b** Acetic acid (AA) extracted and distilled using an alamine and diisobutyl ketone solvent (ADX). Both lignin scenarios are represented in the system boundaries figure. Black dashed line boxes indicate lignin scenario-dependent operations. Lignin can either be burned onsite in the boiler to help produce heat/steam/electricity or sold to a coal power plant and co-fired with coal to produce electricity. If lignin is burned onsite, steam is run through a turbine to produce electricity. If lignin is exported to a coal power plant, no onsite electricity is made and electricity must be purchased from the grid for biorefinery operations. Green boxes highlight product made/energy produced

Cradle to biorefinery exit gate system boundaries are used to evaluate acetic acid production (Fig. 6a, b). A functional unit of 1 tonne of acetic acid produced from a biorefinery system with 21 year operating time frame is used in the analysis. Environmental impacts considered are the 100 year Global Warming Potential (GWP) [18], and Fossil Fuel Use (FFU). FFU is calculated by summing all fossil fuel inputs (coal, natural gas, crude oil) per tonne of acetic acid. Guidelines for conducting a LCA are set by ISO 14040 [21] and 14044 [22] and this research follows the ISO design. LCAs in this research are developed using SimaPro v.8.0. Scenario results are compared to each other as well as to petroleum based acetic acid produced by methanol carbonylation [15]. A sensitivity analysis is conducted to investigate the effect of a decreased acetic acid yield. Additionally system expansion method for co-products is compared to both economic and mass allocation when lignin is exported to a coal burning facility.

The bio-acetic acid life cycles are broken up into 4 sections: feedstock production and harvesting, ancillary chemicals, the biorefinery, and lignin use (co-product scenarios). Feedstock production and harvesting is the same for each biorefinery configuration. Biorefinery process, ancillary chemical inputs, and lignin use will vary depending on the configuration. Descriptions of each life cycle section and allocation methods for lignin use follow below. System boundary diagrams for each bioconversion pathway are displayed in Fig. 6a, b.

### Feedstock

The feedstock production and harvesting model is supported by operational data from industry (GreenWood Resources, personal communication, 2011–2016), literature [23], and LCA databases [15, 24]. It is the same feedstock model used in [4] and is discussed in more detail in that publication. A brief description is provided here. The feedstock production and harvest model is representative of a coppice harvest system, with the poplar trees being coppiced every 3 years for 6 cycles. The model includes all necessary site preparation, nursery operations, management of the poplar tree stands, harvest operations, and stump removal. Nitrogen fertilizer is applied in the spring following a harvest at a rate of 56 kg N per application. N_2_O emissions from fertilizer and decaying biomass are calculated using the Farm Energy Analysis Tool [25]. Storage of carbon in the harvested poplar biomass as well as in below ground biomass (stump and roots) is included. The amount of carbon stored within the below ground carbon stores is assumed to be the same as willow SRWCs and no change in soil carbon down to a depth of 45 cm is expected to occur during tree growth [26]. The equivalent amount of CO_2_ stored in the poplar wood is calculated using the stoichiometric relationship of CO_2_ to carbon of 3.66 kg kg^−1^and a carbon mass fraction of 51.7% dry wood weight [27] (Table 2a & b). Direct land use change is included using the assumption that fallow land will be used for poplar plantations. Direct land use change associated with establishing the plantation is calculated using the Forest Industry Carbon Assessment Tool v.1.3.1.1. Indirect land use change is excluded from the system boundaries due to uncertainty associated with these models [28]. A transportation distance of 100 km roundtrip is assumed to transport the harvested poplar biomass to the biorefinery gate. In total the feedstock production and harvest model covers a 21 year timespan [4].

### Biorefinery

Currently no commercial facilities are using an acetogen fermentation pathway to produce biofuels and biochemicals. To assess the conversion impacts ASPEN-Plus v.8.6 chemical engineering software is used to simulate potential biorefinery process designs. The acetogen fermentation pathway ASPEN simulation is based on a combination of the NREL model [1], a proposed acetogen fermentation process [29], and laboratory work at the Biofuels and Bioproducts Laboratory at the University of Washington. The simulated biorefinery is assumed to operate on 250,000 tonnes of bone dry biomass per year.

Regardless of the product recovery method used (EAX or ADX), biorefinery operations begin with the same processes; dilute acid pretreatment, enzymatic hydrolysis, and fermentation. Pretreatment, hydrolysis, and fermentation conditions are presented in Table 4. These steps are based on National Renewable Energy Laboratory (NREL) corn stover model, but modified to use a poplar feedstock [1]. Following enzymatic hydrolysis, glucose and xylose are fermented to acetic acid using *Moorella thermoacetica*. The streams exiting the fermentation stage include a solid and liquid stream. Descriptions of the fate of these streams are described below. Incorporated into the biorefinery designs, and included in the LCAs is a wastewater treatment system. The WWT design is based on Humbird et al. [1]. Wastewater streams are treated in aerobic and anaerobic environments to produce clean process water, sludge, and methane. The sludge and methane are sent to the boiler. Solid waste produced from the biorefineries is comprised of ash from the boiler, which is collected and sent to a landfill for disposal.

**Table 4:** Process parameters for pretreatment, enzymatic hydrolysis, and fermentation

Processing step	Process parameter	Value
Pretreatment	H_2_SO_4_ charge (gram of acid per gram of bone dry biomass)	0.011
	Temperature (^o^C)	200
	Xylan to xylose conversion (%)	75
Saccharification	Temperature (^o^C)	50
	Enzyme loading (miligram protein per gram cellulose)	20
	Cellulose to glucose conversion (%)	89
Fermentation	Fermentation temperature (^o^C)	58
	Glucose to acetic acid conversion (%)	92
	Xylose to acetic acid conversion (%)	92

The liquid stream exiting the fermentation is 5 wt % acetic acid and water. To be marketable, acetic acid must be concentrated to 99.8 wt % (glacial acetic acid). When acetic acid concentrations are low (0.5–5 wt %) direct distillation of acetic acid from water is inefficient and liquid–liquid extraction (LLE) is the preferred acetic acid recovery method [12]. In this research two LLE methods, both achieving acetic acid yields of 532 kg per bone dry tonne of biomass, are investigated to purify acetic acid. The first method uses ethyl acetate for extraction followed by distillation to recover the ethyl acetate (EAX). The second LLE method uses an alamine/DIBK extraction (ADX). These two extraction scenarios are described below. Major inputs and outputs for the biorefinery scenarios are presented in Table 5.

**Table 5:** Major inputs and outputs from the biorefinery of each scenario

Input	EAX OC	EAX LE	ADX OC	ADX LE
Feedstock (bone dry) (t)	1.9	1.9	1.9	1.9
Enzymes (kg)	16	16	16	16
Sulfuric Acid (kg)	34	34	34	34
Ammonia (kg)	23	23	23	23
Sodium hydroxide (kg)	45	45	45	45
Clarifier polymer (kg)	1.1	1.1	1.1	1.1
Fermentation nutrients (kg)	50	50	50	50
Lime (kg)	26	0	26	0
Natural gas (GJ)	53	58	19	28
Ethyl acetate (kg)	5.2	5.2	NA	NA
Alamine (kg)	NA	NA	1.9	1.9
Diisobutyl ketone (DIBK) (kg)	NA	NA	1.9	1.9
Electricity (kwh)	0	320	0	300
Output				
Acetic acid (t)	1	1	1	1
Lignin (bone dry) (kg)	0	440	0	440
Electricity (kwh)	2600	0	860	0
CO_2_ (from lignin) (t)	1.8	0	1.8	0
CO_2_ (from natural gas) (t)	2.7	3.2	0.99	1.4

Basis is 1 metric ton of acetic acid

Extractants for each pathway (ethyl acetate for EAX, and alamine and diisobutyl ketone for ADX) are reported for their initial application rates per tonne of acetic acid. It is assumed that these extractants are reused at a 99% recycling rate

*EAX* ethyl acetate extraction, *ADX* alamine/diisobutyl ketone extraction, *OC* onsite combustion of lignin, *LE* Lignin exported to a coal burning power plant

### Ethyl acetate extraction

An overview of the ethyl acetate extraction (EAX) process is presented in Fig. 6a. Following fermentation a mixture of water acetic acid (5% acetic acid by weight) is sent to a liquid–liquid extractor. Here it is mixed with ethyl acetate (EA) and the EA solubilizes the acetic acid. A mixture of acetic acid, EA, and a small amount of water are sent to a dehydration column operating at 118^O^C. EA and the remaining water are distilled off and glacial acetic acid (99.8% acetic acid) is produced. Water and EA are sent to stripping column to recover the EA. EA exiting the stripping column is recycled back to the extractor. Water is sent to an onsite wastewater treatment facility before being cycled back through the process. Major inputs and outputs from the biorefinery are listed in Table 5.

### Alamine/Diisobutyl ketone extraction

An overview of the alamine/DIBK solvent extraction (ADX) process is presented in Fig. 6b. After fermentation, acetic acid in water (5% acetic acid by weight) is sent to an extractor. Acetic acid and water are mixed with alamine and DIBK. Acetic acid combines with alamine and DIBK and is removed from the water. This mixture is sent to a dehydration column (174 °C) to remove any residual water. Following dehydration the mixture of acetic acid, alamine, and DIBK is sent to a stripping column (190 °C). Alamine is removed and a mixture of acetic acid and DIBK is sent to a second stripping column (168 °C). DIBK is removed and glacial acetic acid is produced. DIBK and alamine are recovered and are recycled back into process. Water removed during extraction/distillation is sent to wastewater treatment before being reused. Major inputs and outputs from the biorefinery are listed in Table 5.

In both extraction methods the solid stream separated out after the fermentation stage consists of lignin and other unfermented carbohydrates. To recover these solids and remove some of the residual water, the solid streams are filter pressed to 50% solids. Following concentration of the solids two potential downstream options for the solid stream are evaluated in this study. These are described in more detail below.

### Onsite lignin combustion

Option one consists of combusting lignin onsite to produce heat/steam for the biorefinery operations and producing electricity by running high pressure steam through a steam turbine; using the moderate pressure steam exiting the turbine for the process. This practice is common in proposed biofuel biorefinery designs [1, 4] and pulp mills [30]. Compared to second generation lignocellulosic ethanol production, producing glacial acetic acid requires more heat/steam and combusting lignin alone cannot meet the entire energy demand. Extraction/distillation of acetic acid requires a significant amount of steam. To meet this demand, natural gas is imported and combusted with the lignin. To reach the temperatures needed for extraction/distillation moderate pressure steam would be required. However, technoeconomic work with the ASPEN model identified an economic benefit to instead first create high pressure steam and pass it through a turbine to produce moderate and low pressure steam. The conversion of high pressure steam to lower pressure steam through the turbine generates an amount of electricity that exceeds the needs of the biorefinery. The conversion efficiency of heat to steam is assumed to be 80% for both natural gas and lignin. The excess electricity can be sold to the electrical grid to increase the revenue generated from the biomass. The production of excess electricity is greater in the ethyl acetate extraction process as this method has a greater steam demand, and therefore, more high pressure is passed through the turbine.

For the LCA of the onsite lignin combustion scenario, the electricity by-product is treated using system expansion per ISO standards [22]. The electricity by-product meets the requirements for using system expansion as it is currently produced from other sources and life cycle data for the production of electricity from these other sources can be obtained [31]. System expansion is the most common method used in biofuel LCAs to deal with an excess electricity by-product [32]. It is assumed that the electricity will be sold to the grid and displace electricity produced from natural gas, a likely candidate for the marginal electricity source [33]. An avoided production credit is generated for displacing this fossil fuel source of electricity with electricity produced from a renewable source. Fugitive emissions from process operations are estimated to be 2% of unit process flows [34].

### Sell lignin to power plant

The second option for lignin is to export it to a coal power plant and co-fire with coal. This has been shown to be a viable option and can economically and environmentally benefit both the biorefinery and the coal power plant [17]. In this scenario lignin is considered a co-product to acetic acid production. It is dried to about 50% lignin by weight (50% water) and shipped to a nearby coal power plant and used in place of coal. The amount of coal displaced is based on the energy content of the lignin. The moisture content of the lignin will affect the energy content and must be accounted for when calculating the amount of coal displaced (i.e., the energy required to remove water prior to combustion is included in the coal displacement calculation). To determine the coal displacement by selling the lignin as a co-product, the HHV of wet lignin (50% MC) was estimated using ASPEN. Lignin was modeled as vanillin C_8_H_8_O_3_ with an HHV of 25.2 MJ/kg [1], similar to the experimental value of dilute acid pretreated lignin of 21.4 MJ/kg [35]. Assumed HHVs for all combustible materials are reported in Table 6. Exporting lignin as a co-product requires that other forms of energy must be used to meet the needs of the biorefinery. Natural gas is assumed to be combusted at the biorefinery to provide heat and steam. In this scenario it is assumed that a lower pressure boiler is used and the additional expense of a turbine to generate power would not be incurred. Natural gas boilers are more commonly used in the industry due to the relatively low capital cost in the range of $8–$23/kW [36] and their relatively small physical size. In contrast, biomass boilers are larger in size and have high capital cost ($94–$125/kW) [37] due to more complex design. Consequently, natural gas boilers are typically less expensive than those that would be suitable for combusting lignin. Exporting lignin and using natural gas as the sole driving fuel represents a lower cost alternative. A high pressure boiler with turbo-generator would not be appropriate in such a biorefinery design approach. For the lignin export case the biorefinery electricity needs are assumed to be met by importing electricity from the U.S. national grid.

**Table 6:** High heating values (HHVs) for lignin, coal, and natural gas

Material	High heating value (HHV) (MJ/kg of material)
Lignin	25.2
Natural gas	54.4
Coal	26.2

### Ancillary chemicals

Biorefinery operations require chemical inputs to convert the poplar biomass to acetic acid. The production of these chemicals is grouped into the ancillary chemicals section. Unit process data for the chemical inputs come from the USLCI [15], EcoInvent [24], literature, and the private sector. The electricity source in each unit process is set to come from a unit process representative of the 2012 U.S. national grid [38]. Data for enzyme production is supplied from Novozymes for their Cellic Ctec3 cellulases [39]. Transportation distances for each chemical are determined using the 2007 U.S. commodity flow survey [40].

### Allocation and sensitivity analysis

System expansion is used in evaluating the base case for the four bio-acetic acid production scenarios. As discussed above, this is deemed to be the appropriate treatment of the life cycle impacts for the product and excess electricity/lignin co-product. However, the results are also evaluated using mass and economic allocation methods to determine the life cycle effect of allocating life cycle impacts between acetic acid production and the lignin co-product (in the lignin exporting scenarios). Allocating the life cycle impacts between acetic acid and lignin divides the environmental benefits (i.e., carbon sequestration) and burdens (i.e., natural gas combustion) between acetic acid and the lignin co-product. To account for the movement of carbon within the biorefining systems the carbon sequestered in the poplar biomass is allocated to either acetic acid (i.e., glucose and xylose) or lignin. Producing one tonne of acetic acid requires 1.8 tonnes of poplar biomass (dry weight). At a 51.7% carbon content [28], 1.8 tonnes of poplar contain 930 kg of carbon. Through the bioconversion process 400 kg of this carbon will go into the acetic acid. 380 kg of the carbon is contained in the lignin. The 150 kg of carbon remaining in the system (carbohydrates in liquid streams) is divided between the acetic acid product and lignin co-product. The amount of this 150 kg assigned to either the acetic acid product or lignin co-product depends on the allocation method being assessed (mass or economic). From the acetic acid product view point, allocation also removes all processes that are downstream of the biorefinery–and tied to the lignin co-product–from the life cycle production of acetic acid; including coal displacement and emissions from lignin combustion at the coal burning facility.

The mass allocation approach divides the life cycle processes amongst acetic acid and lignin according to the mass of each product. For every tonne of acetic acid produced, 394 kg of lignin (dry weight) is exported. Economic allocation divides life cycle processes amongst acetic acid and lignin based on the economic values of these two products. The minimum selling price was calculated in ASPEN and used to establish the value of the acetic acid. In the EAX LE scenario techno-economic analysis identified the minimum selling price of acetic acid to be $819 per tonne and economic value of the lignin exported to the coal facility to be $39 per tonne (assuming $4.40 per MMBTU). In the ADX LE scenario the selling price for acetic acid is $677 per tonne and the value of the exported lignin to be $39 per tonne.

A sensitivity analysis is conducted to test for model sensitivity to changes in fermentation yields. The fermentation yield of glucose to acetic acid in this research is set at 92%. Maintaining a fermentation yield of 92% may be difficult when operating at commercial scale and could likely fluctuate. If the fermentation yield decreases the amount of acetic acid produced would decrease and the amount of unfermented carbohydrates, and therefore, the amount of biomass available to burn would increase. To test the effect of a decreased fermentation yield and to evaluate model sensitivity, a simulation is performed for the EAX OC and EAX LE scenarios in which the fermentation yield is decreased by 10%. Only EAX is tested for sensitivity analysis as this system is more likely to be commercialized and it is expected that the effect of a decreased acetic acid yield would be similar for both EAX and ADX.

## Data Availability

No supporting information is provided.

## Abbreviations

ADX: Alamine/diisobutyl ketone extraction
DIBK: Diisobutyl ketone
EAX: Ethyl acetate extraction
FFU: Fossil fuel use
GHGs: Greenhouse gases
GWP: Global warming potential
LE: Lignin exportation
LCA: Life cycle assessment
OC: Onsite combustion
